# Management of Autoimmune Status Epilepticus

**DOI:** 10.3389/fneur.2018.00259

**Published:** 2018-05-09

**Authors:** Batool F. Kirmani, Donald Barr, Diana Mungall Robinson, Zachary Pranske, Ekokobe Fonkem, Jared Benge, Jason H. Huang, Geoffrey Ling

**Affiliations:** ^1^Epilepsy Center, Baylor Scott and White Health Neuroscience Institute, Temple, TX, United States; ^2^Texas A&M Health Science Center, College of Medicine, Temple, TX, United States; ^3^Department of Psychiatry, University of Virginia, Charlottesville, VA, United States; ^4^Baylor University, Waco, TX, United States; ^5^Department of Neurosurgery, Baylor Scott and White Health Neuroscience Institute, Temple, TX, United States; ^6^Division of Neuropsychology, Baylor Scott and White Health Neuroscience Institute, Temple, TX, United States; ^7^Uniformed Services University of the Health Sciences, Bethesda, MD, United States

**Keywords:** autoimmune encephalitis, status epilepticus, autoimmune antibodies, neuroimmunomodulatory therapies, autoimmune epilepsy

## Abstract

Status epilepticus is a neurological emergency with increased morbidity and mortality. Urgent diagnosis and treatment are crucial to prevent irreversible brain damage. In this mini review, we will discuss the recent advances in the diagnosis and treatment of autoimmune status epilepticus (ASE), a rare form of the disorder encountered in the intensive care unit. ASE can be refractory to anticonvulsant therapy and the symptoms include subacute onset of short-term memory loss with rapidly progressive encephalopathy, psychiatric symptoms with unexplained new-onset seizures, imaging findings, CSF pleocytosis, and availability of antibody testing makes an earlier diagnosis of ASE possible. Neuroimmunomodulatory therapies are the mainstay in the treatment of ASE. The goal is to maximize the effectiveness of anticonvulsant agents and find an optimal combination of therapies while undergoing immunomodulatory therapy to reduce morbidity and mortality.

## Introduction

Status epilepticus (SE) is defined as a condition resulting either from the failure of the mechanisms responsible for seizure termination or from the initiation of seizure inducing mechanisms, which can lead to abnormally prolonged seizures (5 min in the case of convulsive SE) (1). In more prolonged cases (greater than 30 min) (2, 3), long-term neurological injury such as neuronal death, injury, and alteration of cerebral networks can occur. The annual incidence rate of SE is 12.6 per 100,000 worldwide and 11.7 per 100,000 in developed countries (4).

While common causes of SE seen in the ICU (including stroke, infection, and subtherapeutic antiepileptic drug levels in patients with epilepsy) are often readily identified (5), a less common cause of SE is autoimmune status epilepticus (ASE). In these patients, autoantibodies against cell surface or synaptic proteins disrupt receptors or voltage-gated ion channels, resulting in rapidly progressive encephalopathy, abnormal movements, psychiatric symptoms, and often recurrent seizures. Some of these patients with autoantibodies and new-onset seizures—*autoimmune epilepsies*—will develop convulsive or non-convulsive ASE (6). There is a broad range of auto antigens in autoimmune encephalopathies with seizures both among those with intracellular antigens (GAD, Hu, DNER, Sox1 Ampiphysin, Ma, and CV2) and surface antigens (LG1, CASPR2, NMDAR, GlyR, AMPAR, GABA_B_R, mGluR5, DPPX, GABA_A_R, and Neurexin-3a). These patients can present with antiepileptic drug resistant epilepsy; however, potential for reversal with immunotherapy and early treatment has shown improved survival and cognition (7).

Autoimmune status epilepticus is a rare condition, although its outcomes can be profound. In their review of 1,111 cases of autoimmune SE, Holzer et al. found the prognosis was bleak for those with ASE. 50% of cases demonstrated major morbidity or mortality, with 16% of patients dying and 35% of patients surviving with significant deficits. Age seemed to be an important factor related to likelihood of fatal outcome in the context of ASE, with those with higher age of onset being associated with greater mortality while those of younger age of onset are generally more likely to recover with major behavioral, psychiatric, or epileptogenic deficits (8). Spatola et al. reported a retrospective study of 570 episodes of SE and found that ASE accounted for just 2.4% of SE episodes. ASE was more common in younger patients (mean age: 44 vs 60 years), was more likely to be refractory to antiepileptic drug treatment, and was associated with lower morbidity (return to baseline conditions in 71%) without any difference in mortality (9). Harutyunyan et al. conducted a retrospective cohort study to characterize the underlying etiologies, clinical symptoms, reasons for ICU admission, and mortality of critically ill patients with autoimmune encephalitis and found that SE was the most common reason for admission. In this study, the median age of patients with autoimmune encephalitis was 55 years (25–87 years, *n* = 16) (10).

## Establishing Etiology and Therapeutic Options in ASE

The presence of antineuronal antibodies against central nervous system proteins is associated with a group of encephalopathies termed autoimmune encephalopathies. They may be categorized based on their antigen target (intraneuronal, surface receptor, or synaptic). Antibodies to cell surface antigens are the most common, considered idiopathic in nature, while the intraneuronal antibodies are more often associated with a neoplasm (11, 12). There are some autoimmune encephalopathies more frequently associated with seizures termed autoimmune epilepsies. These autoimmune epilepsies are seen in patients with antibodies to intracellular antigens (GAD, Hu, DNER, Sox1 Ampiphysin, Ma, and CV2) and surface antigens (LG1, CASPR2, NMDAR, GlyR, AMPAR, GABA_B_R, mGluR5, DPPX, GABA_A_R, and Neurexin-3a); however, there is potential for reversal with immunotherapy, and early treatment has shown a general rate of improved survival and cognition (7).

At disease onset, there is little to distinguish the different types of autoimmune encephalopathies from one another. Subacute development of short-term memory loss is among the key clinical features of autoimmune encephalopathies, along with rapid development of cognitive and working memory impairment, mood changes, and seizures (13). In their proposed diagnostic criteria for possible autoimmune encephalitis, Graus et al. included new focal CNS findings, CSF pleocytosis, MRI features suggestive of encephalitis, and seizures not explained by a previous known seizure disorder (14). Note that their criteria does not rely on antibody testing and response to immunotherapy but rather levels of evidence for autoimmune encephalitis (possible, probable, or definite) leading to earlier treatment with immunotherapy, which can lead to improved survival and cognitive outcomes (14, 15).

Sakusic and Rabinstein found that early manifestations of autoimmune encephalitis are agitation or new behavioral changes mimicking psychosis. They also note that even when CSF and MRI brain are normal, autoimmune encephalitis is still possible. In fact, the clinical presentations can vary with the same antibodies (16).

Bien and Holtkamp found that traits associated with an increased likelihood of autoimmune epilepsy included young women (15–45 years old), history of autoimmune conditions, epilepsy onset together with prominent psychiatric or cognitive impairment, and unexplained new-onset epilepsy in adults with SE or high seizure frequency (7). Electroencephalogram findings are generally non-specific in distinguishing between autoimmune epilepsies, with the exception of extreme delta brush which is seen in anti-NMDAR encephalitis (17). CSF pleocytosis is seen in majority of autoimmune epilepsies except LG1. Some seizure types can support specific autoimmune epilepsies such as faciobrachial dystonic seizures (seen in patients with LGI1 antibodies) and pilomotor seizures (seen with LGI1, Hu, and Ma antibodies).

In general, a broad spectrum of antibody testing is recommended to avoid delays that may be caused by isolated single antibody testing. Encephalopathy with seizures manifesting as rapidly evolving cognitive impairment with neuropsychiatric features or focal neurological deficits may be related to several antibodies, with NMDAR and LGI1 among the most likely. Furthermore, it is noted that the patients with voltage-gated potassium channel complex antibodies respond well and can even become seizure free with immunotherapy. Patients with faciobrachial dystonic seizures and LI1 antibodies respond well to steroids but had little response to AEDs (18). This is particularly challenging with new-onset temporal lobe seizures which are frequently accompanied by various degrees of psychiatric associations (such as depressed mood or memory deficits, for example) but may well be the onset of anti-NMDAr encephalitis (19).

There have been relatively few studies of the exact pathophysiology of ASE and even sparser characterization of outcomes related to particular pathologies. Bien found that patients with autoimmune epilepsy who presented with SE or very high seizure frequency, most cases were associated with NMDAR, LGI1, GABABR, GABAAR, and GAD antibodies (18).

In terms of treatment, corticosteroids, immunosuppressive treatments, intravenous immunoglobulin (IVIG), etc., assume that inflammation is the underlying cause of SE in patients. However, there is only limited evidence to support these treatment options. Some clinical studies have linked systemic inflammation to epilepsy; however, it is not clear from these trials alone whether inflammation directly causes SE. Children with febrile seizures have elevated levels of IL-1B, IL-6, and HMGB1, which are all inflammatory markers (18). Furthermore, histopathological findings from temporal cortex from patients with new-onset focal seizures that progressed to SE show gliosis of both astrocytes and microglia, strong IL-1B expression, and sparse lymphocytic infiltration, again suggestive of an inflammatory pathology. In bench studies, induced SE is linked to a multitude of inflammatory cascades in animal models. Within 30–60 min of SE onset, IL-1B, TNF-a, and IL-6 transcript levels are increased for 24–72 h In compromised brains, these changes are associated with a more severe acute course and worse long-lasting neuropathological symptoms. It appears that inefficient endogenous anti-inflammatory control in the brain may be characteristic of chronic recurrent seizures and SE (18).

Despite controversy as to the exact mechanisms, limited clinical data suggest patients with known or suspected autoimmune encephalitis may still benefit from immunotherapy (18). Response to immunotherapy is commonly delayed, so caution to avoid overtreatment is advised. As with Holzer et al., first line treatments with corticosteroids, IVIG or plasma exchange may be effective, but refractory cases may require aggressive treatment regimens that may have significant side effects (8). Rituximab, cyclophosphamide, mycophenolate mofetil, tacrolimus, as well as VNS have been used in refractory cases (19–23). In their comprehensive review, Bien and Holtkamp provided an immunotherapy algorithm for conditions associated with antibodies against cell surface receptors, largely derived from reported experience with anti-NMDAR encephalitis. These include corticosteroids, IVIG, independent plasma exchange, or a combination of these symptoms (7).

Early implementation of immunomodulatory treatment may play a role for survival and better cognitive functions. In their retrospective review of 27 patients with suspected autoimmune epilepsy who underwent a 6- to 12-week trial of IV methylprednisolone, IV immune globulin, or both, Toledano et al. found that 18 patients responded with at least a 50% seizure reduction frequency, including 10 who became seizure free. 52% improved with the first agent, and of those who required a second agent, 43% responded. In addition, they found favorable response correlated with shorter interval between symptom onset and treatment initiation, and response was sustained in 11 of 13 responders followed for more than 6 months after initiating long-term oral immunosuppression with either mycophenolate mofetil or azathioprine. The authors conclude that these findings justify consideration of a trial of immunotherapy in patients with suspected autoimmune epilepsy (15).

Zeiler et al. conducted a literature review of 22 articles encompassing 27 adult patients to determine the effectiveness of plasmapheresis for refractory SE in adults. Inclusion criteria were all prospective and retrospective studies of any size, adult human subjects (≥18 years old), and use of plasmapheresis for the purpose of seizure control in the setting of refractory SE. There was a wide variety of etiologies of SE/RSE, including: unknown suspected autoimmune (*N* = 8 pts), NMDA receptor encephalitis (*N* = 6 pts), anti-GAD (*N* = 3 pts), Hashimoto’s encephalitis (*N* = 2 pts), and one patient each with anti-VGCC encephalitis, anti-VGKC encephalitis, anti-SSA encephalitis, anti-GABA encephalitis, herpes simplex virus encephalitis, Creutzfeldt–Jakob disease, Rasmussen’s encephalitis, and new-onset refractory SE. The most common types of seizures were generalized refractory SE (*N* = 15 pts), followed by focal refractory SE and non-convulsive SE (*N* = 6 pts each). The mean duration of treatment before starting plasmapheresis therapy was 16.2 days. The mean number of AEDs before plasmapheresis was 6.5 (18 studies; range 2–11). Other immunotherapies including corticosteroids, IVIG, and rituximab were used in most studies (21 of 22 studies). 13 patients (48%) had complete resolution of seizures, 13 patients (48%) had no response, and 1 patient (4%) had partial temporary electroencephalogram-based response. Patients with generalized refractory SE were the most likely to respond (67%, 10 of 15 patients) followed by refractory SE and non-convulsive SE (both = 33%, 2 of 6 patients). After plasmapheresis, 29% of patients who initially responded (4 of 14 patients) had seizure recurrence. Given these findings, the authors concluded that plasmapheresis as a treatment for RSE could not be recommended at this time due to patients having an uncertain response (24).

Febrile infection-related epilepsy syndrome (FIRES) is the also most catastrophic immune mediated epileptic encephalopathy which affects healthy children. The spectrum of this rare clinical illness is unknown but preceding febrile infection points toward immune etiology which is triggered by infection (25). Neuroinflammation has been shown to play a role in experimental animal models (26). The diagnosis is clinical and ranges from seizure clusters to refractory SE preceding febrile illness (25).

Conventional therapies with antiepileptic medications often fail, and early treatment with ketogenic diet, immune-modulators, and cannabidiol may help reduce seizure burden, but evidence remains anecdotal (27–29). Ketogenic diet is also utilized in other forms of super-refractory SE, both in adults and children, particularly in suspected immune related cases. Ketogenic diet can be administered parenterally and has antiepileptic effect through direct inhibition of the postsynaptic excitatory AMPA receptor by decanoic acid (30). There is also evidence that it has anti-inflammatory role which can be hypothesized as being useful in the treatment of FIRES and other related disorders (31).

## Future Directions

Autoimmune status epilepticus is an infrequently observed but potentially devastating disorder with a multitude of clinical presentations depending upon the type of antibodies. During the recent years, a link has been established between various antibodies and their specific presentation Researchers continue to find new antibodies but as the available testing for antibody-mediated encephalitis expands, but physicians have limited data regarding optimal treatment regimens as it pertains to acute intervention in patients. IV immunoglobulin and plasmapheresis are also commonly included as first line therapies. Corticosteroids are also used in treatment; however, dosing, duration and threshold for response time before changing to alternative therapy remains unclear. Physicians must continue to work toward early recognition and effective interventions in patients presenting with ASE as well as understanding the underlying pathophysiology to better tailor intervention and more basic research is needed to recognize biomarkers to develop novel therapeutic agents for this life threatening condition.

## Author Contributions

All the authors have contributed equally to this work.

## Conflict of Interest Statement

The authors declare that the research was conducted in the absence of any commercial or financial relationships that could be construed as a potential conflict of interest.
